# Securing jugular central venous access devices with dressings fixed to a liquid adhesive in an intensive care unit population: a randomised controlled trial

**DOI:** 10.1186/s13063-022-06322-9

**Published:** 2022-05-12

**Authors:** India Pearse, Amanda Corley, Emily N. Larsen, Claire M. Rickard, Robert S. Ware, Jill Campbell, Fiona Coyer, Evan Alexandrou, Catherine O’Brien, Nicole Marsh

**Affiliations:** 1 Nursing and Midwifery Research Centre, Royal Brisbane and Women’s Hospital, Herston, Australia; 2 Critical Care Research Group, The Prince Charles Hospital and University of Queensland, Chermside, Australia; 3 School of Nursing and Midwifery, Griffith University, Brisbane, Australia; 4 School of Nursing, Midwifery and Social Work, University of Queensland, Brisbane, Australia; 5 AVATAR Group, Griffith University, Brisbane, Australia; 6 Patient-Centred Health Services, Menzies Health Institute Queensland, Southport, Australia; 7 Herston Infectious Diseases Institute, Metro North Hospital and Health Service, Herston, Australia; 8 Menzies Health Institute Queensland, Griffith University, Brisbane, Australia; 9 National Health and Medical Research Council Centre for Research Excellence, Menzies Health Institute Queensland, Griffith University, Brisbane, Australia; 10 Intensive Care Services, Royal Brisbane and Women’s Hospital, Herston, Australia; 11 School of Nursing, Queensland University of Technology, Brisbane, Australia; 12 Centre for Healthcare Transformation, Faculty of Health, Queensland University of Technology, Brisbane, Australia; 13 School of Nursing and Midwifery and Centre for Applied Nursing Research, Western Sydney University, Penrith, Australia; 14 Department of Intensive Care, Liverpool Hospital, Liverpool, Australia

**Keywords:** Central venous access device, Dressing failure, Intensive care unit

## Abstract

**Background:**

Central venous access devices (CVADs) can have high rates of failure due to dressing-related complications. CVADs placed in the internal jugular vein are at particular risk of dressing failure-related complications, including catheter-associated bloodstream infection and medical adhesive-related skin injury. Application of Mastisol liquid adhesive (MLA) may reduce CVAD dressing failure and associated complications, by reducing the frequency of dressing changes. The aim of this study is to investigate whether, in an intensive care unit (ICU) population, standard dressing care with or without the addition of MLA, improves internal jugular CVAD dressing adherence.

**Methods:**

This two-arm, parallel group randomised controlled trial will be conducted in three Australian ICUs. A total of 160 patients (80 per group) will be enrolled in accordance with study inclusion and exclusion criteria. Patients will be randomised to receive either (1) ‘standard’ (in accordance with local hospital policy) CVAD dressings (control) or (2) ‘standard’ dressings in addition to MLA (intervention). Patients will be followed from the time of CVAD insertion to 48 h after CVAD removal. The primary outcome is ‘dressing failure’ defined as requirement for initial CVAD dressing to be replaced prior to seven days (routine replacement).

**Discussion:**

This study will be the first randomised controlled trial to evaluate the clinical effectiveness of MLA in the adult intensive care unit population and will also provide crucial data for patient-important outcomes such as infection and skin injury.

**Trial registration:**

Australian New Zealand Clinical Trials Registry ACTRN12621001012864. Registered on 2 August 2021

## Administrative information

**Table:** 

Title	Securing jugular central venous access devices with dressings fixed to a liquid adhesive in an intensive care unit population: a randomised controlled trial
Trial registration	ACTRN12621001012864
Protocol version	Version 1.0, dated 13 May 2021
Funding	Eloquest Healthcare
Author details	India Pearse, Royal Brisbane and Women’s Hospital Amanda Corley, Griffith University/Royal Brisbane and Women’s Hospital Emily Larsen, Griffith University/Royal Brisbane and Women's Hospital Claire Rickard, University of Queensland Robert S Ware, Griffith University Jill Campbell, Griffith University Fiona Coyer, Queensland University of Technology/Royal Brisbane and Women’s Hospital Evan Alexandrou, Liverpool Hospital Catherine O’Brien, Royal Brisbane and Women’s Hospital Nicole Marsh, Royal Brisbane and Women’s Hospital
Name & contact information for the trial sponsor	Prof Nicole Marsh University of Queensland nicole.marsh@health.qld.gov.au
Role of sponsor	The study database will be hosted on the sponsor’s server, however, the sponsor will not have any input into study design, data collection, analysis, interpretation of the data or manuscript preparation.

## Introduction

### Background and rationale

Central venous access devices (CVADs) are used extensively worldwide to deliver critical treatment and haemodynamic monitoring [1, 2]. They are a frequently consumed medical device in hospitals, with more than 130,000 used in Australian public hospitals each year [3]. CVADs are inserted into large veins of the neck, chest, arm or groin and terminate in the central vasculature [1, 4]. Despite their utility, these important medical devices frequently result in complications, with reported device failure of up to 30% due to complications such as dislodgement, blockage/occlusion, fracture/breakage, thrombosis, pleural/pericardial effusion or infection [1, 2, 5]. These complications typically result in delayed treatment due to the need for reinsertion, causing extended admission time, increased healthcare costs, venous depletion and increased morbidity and mortality [6].

Although the placement of CVADs in the jugular vein has been associated with an increased risk of central line associated bloodstream infection (CLABSI) and device failure compared to placement in the subclavian vein [2, 7, 8], the jugular vein remains a frequently selected site for CVAD placement in intensive care units (ICUs). This device failure may be attributed, in part, to inadequate dressing of the CVAD, resulting in (1) catheter dislodgement and (2) micromotion leading to complications such as occlusion, venous thrombosis, catheter rupture and infection [9]**.**

Traditional practice for dressing and securement of internal jugular (IJ) CVADs has been the use of polyurethane transparent dressings with or without additional securement from sutures or commercial sutureless stabilisation devices [9]. However, current dressing and securement practices are often inadequate, as IJ CVAD dressings frequently fail as a result of diaphoresis, beard growth, multi-vector pull forces related to the ‘drag’ of multiple infusion lines, intermittent ‘catching’ on objects and increasingly early mobilisation of ICU patients [2, 10, 11]. Each time a dressing fails it requires replacement, therefore placing patients at a higher risk of medical adhesive-related skin injury (MARSI) [12]. CVAD-related MARSI typically includes bruising, local site infections, pressure injuries, maceration, dermatitis, and mechanical injuries such as skin tears and blisters which have the potential to be disfiguring and lead to CVAD failure [13, 14]. Furthermore, dressing failure creates an opportunity for skin bacteria to contaminate the catheter insertion site leading to CLABSI or local infection, with a threefold increased risk of CLABSI as a result of skin colonisation and the migration of organisms down the catheter following disruption of the second dressing [15].

Mastisol liquid adhesive (MLA) (Eloquest Healthcare, Inc., Ferndale, MI) is a non-water-soluble gum mastic liquid adhesive designed to improve dressing adherence and integrity [12] and consequently decrease dressing failure. MLA is applied to the skin under the dressing after skin antisepsis. The purpose of MLA is to reduce frequent and often painful dressing changes and the resulting incidence of both CLABSI and MARSI [12], by improving dressing adhesion. A recent study in healthy volunteers found no statistical difference in bacterial growth with the use of MLA compared to standard care alone, supporting its safe use in this patient population [12]. Furthermore, a recent audit of IJ CVADs dressings in a cardiovascular ICU found an improvement with dressing adhesion from 11% (*n* = 4) to 100% (*n* = 30) with the use of MLA [16]. However, despite being in clinical use in Australia for many years, MLA has not been tested rigorously in a randomised controlled trial (RCT). To determine if MLA is a clinical and cost-effective intervention to improve IJ CVAD dressing adherence, we propose a multi-centre, two-arm, parallel group RCT.

### Objectives

The objectives of this study are to:

1. Assess the effectiveness of MLA, compared to ‘standard’ dressing care, in reducing number of dressing changes, adverse events, clinician workload and costs in associated with IJ CVADs
2. Evaluate the acceptability of MLA to adult ICU patients and clinicians

### Trial design

The trial design is as follows: multi-centre, superiority, two-arm, parallel group RCT.

## Methods: participants, interventions and outcomes

### Study setting

This study will be undertaken at the following tertiary-level academic teaching hospitals in Australia:

1. The Prince Charles Hospital, Chermside, Queensland
2. Logan Hospital, Meadowbrook, Queensland
3. Liverpool Hospital, Liverpool, New South Wales

### Eligibility criteria

Patients will be eligible for inclusion in the study if they are

1. ≥ 18 years old
2. Expected to require an IJ CVAD for ≥ 72 h
3. Require ≥ 24 h treatment in the ICU
4. Screened within 12 h of CVAD insertion

Patients will be excluded if they

1. Have a CVAD inserted under emergency conditions
2. Have a current bloodstream infection diagnosed within 24 h prior to CVAD insertion,
3. Have a pre-existing concurrent CVAD expected to dwell for > 24 h
4. Are receiving end-of-life care
5. Have previously been enrolled in the study

### Who will take informed consent?

Research staff will screen patients according to inclusion and exclusion criteria. If eligible, patients will be approached by research staff for informed consent. If the patient does not have capacity to provide consent, the patient’s substitute decision maker will be approached. If prospective consent is not able to be obtained from either the patient or their substitute decision maker within the inclusion criteria time frame, the patients will be enrolled in the trial under a ‘consent to continue’ model as approved by the reviewing Human Research Ethics Committee (HREC). The patient and/or their substitute decision maker will then be approached for consent either in person (preferred) or by phone as soon as possible after enrolment.

### Additional consent provisions for the collection and use of participant data and biological specimens

Not applicable.

### Interventions

#### Comparators

- Control: ‘Standard’ CVAD dressings as per individual hospital policy
- Intervention: ‘Standard’ CVAD dressings in addition to MLA

### Intervention description

#### Control group

The control group will have their CVAD dressed and secured in accordance with standard hospital policy [17–19] and the individual needs of the participant.

#### Intervention group

The intervention group will have their CVAD dressed and secured in accordance with standard hospital policy, plus the use of MLA immediately prior to dressing application (all other products, including skin barrier products and sutures are permitted with MLA). MLA will be applied using the applicator as per manufacturer’s instructions (i.e. a single-layer border under the expected perimeter of the CVAD dressing).

### Criteria for discontinuing or modifying allocated interventions

Criteria for discontinuation or modification of interventions will be:

1. Development of MARSI attributed to MLA, as ascertained by each site’s principal investigators and reviewed by the HREC
2. Development of CLABSI attributed to MLA, as ascertained by each site’s principal investigators and reviewed by the HREC

If a patient develops an adverse reaction thought to be the result of MLA, use of MLA will cease and the HREC, project manager and coordinating principal investigator notified.

### Strategies to improve adherence to interventions

Standardised education will be provided to both research and clinical staff at each site prior to study commencement to improve adherence to study interventions. Research staff will, where possible, be present for the first application of MLA on each patient and provide ongoing study information and resources to clinical staff responsible for CVAD dressing care throughout the duration of each CVAD dwell. Research staff will be available to be contacted outside of business hours to answer any questions or queries clinical staff may have. If MLA is not applied at the time of dressing change, a protocol deviation will be recorded, and MLA will be reapplied at the next dressing change.

### Permitted and prohibited concomitant cares

All concomitant cares will be permitted whilst the patient is enrolled in the trial. No cares will be prohibited.

### Post-trial care

After the CVAD is removed as clinically indicated, outcome and adverse event data will be collected for a further 48 h by research staff. After this time, no further post-trial care is required.

### Outcomes

#### Primary outcome

Dressing failure: defined as requirement for initial IJ CVAD dressing to be replaced due to the dressing lifting at the edges at any point during CVAD dwell but prior to scheduled dressing change (scheduled dressing changes are every seven days). The requirement for dressing changes will be determined by clinical staff treating the patient, and the clinical indication for dressing change will be recorded. If a patient’s CVAD is removed prior to seven days with the primary dressing still in situ, the dressing will be considered not to have failed.

#### Secondary outcomes

1. All-cause CVAD failure: a composite of failure resulting in CVAD removal. Includes pain, infiltration/extravasation, blockage/occlusion (with or without leakage), fracture, thrombosis, dislodgement (complete or partial), or haematoma
2. Individual types of CVAD failure resulting in CVAD removal [pain, infiltration/extravasation, blockage/occlusion (with or without leakage), fracture, thrombosis, or dislodgement (complete or partial)]
3. ‘Central line associated bloodstream infection’ defined as per the National Healthcare and Safety Network (NHSN) [20]
4. ‘Primary bloodstream infection’ defined as per the NHSN [20]
5. ‘Local infection’ as defined by the Centres for Disease Control and Prevention (CDC)/NHSN ‘Arterial or venous infection’ criteria [21]
6. Loss of dressing integrity not requiring dressing change (i.e. lifting at edges with/without reinforcement required) assessed for all dressings per patient
7. Dressing dwell time (from dressing application to removal in days) assessed for all dressings per patient
8. Premature dressing removal (before seven days from dressing application; all dressings per patient)
9. Number of dressing changes (from first application to last removal; all dressings per patient)
10. Device dwell-time (time from CVAD insertion to removal in hours)
11. Serious adverse events (i.e. mortality, CLABSI, MARSI)
12. Adverse skin events relating to MARSI (e.g. pain, itch, erythema, skin stripping, blister, skin tear, irritant contact dermatitis, maceration, folliculitis or infection) [22]
13. Cost (cost and number of products used, cost of treating complications, staff time for device insertion), as informed by standard diagnosis related groups (DRGs), staff time estimates to apply and remove dressings and product costs.
14. Staff and patient satisfaction on dressing application and removal (0 = not at all satisfied to 10 = completely satisfied) assessed at initial application, all dressing changes and final removal for all dressing changes per patient.
15. Skin colonization, measured both descriptively (i.e. organism) and quantitatively (i.e. colony forming units)

### Participant timeline

Participants are enrolled prior to or within 12 h of their CVAD insertion, and will continue on the study until 48 h after their CVAD is removed. It is anticipated that each CVAD will dwell for an average of 7 days, resulting in an average enrolment time for each participant of 9 days (including CVAD dwell whilst in ICU and on the ward).

### Sample size

A total of 160 patients will be recruited. At 90% power and a significance level of 0.05, 77 patients per group are required to detect a 25% absolute difference in the primary outcome (control failure 50%, intervention failure 25% [2]). To account for potential attrition, three additional patients per group will be recruited.

### Recruitment

Each participating site has been targeted as they have a track record of conducting efficient and accurate research within their ICUs. Site feasibility assessments (i.e. anticipated number of eligible patients, adequate staffing resources, clinical equipoise) were conducted at each site prior to study commencement. Each member of the research team at each site will be provided extensive education on the study protocol prior to recruitment commencement, and will be encouraged to notify the project manager of any recruitment difficulties to ensure strategies are in place to overcome these and ensure adequate participant enrolment.

### Assignment of interventions: allocation

#### Sequence generation

Patients will be randomised in a 1:1 ratio to either ‘standard’ dressing care or ‘standard’ dressing care in addition to MLA. Randomisation will occur in computer-generated randomly varied block sizes of four and six and will be stratified by patient sex to account for facial hair differences.

#### Concealment mechanism

Randomisation allocation will be concealed until the point of randomisation using a central, web-based randomisation service embedded within the study database.

#### Implementation

A statistician independent of the research team will generate the randomisation allocation sequence, and this will be uploaded onto the randomisation service without viewing by the research team. Patients will be enrolled and randomised by members of the research team, with intervention allocation assigned as per the randomisation service.

### Assignment of interventions: blinding

#### Who will be blinded

Due to the nature of the intervention, blinding of patients/clinicians and research staff to the intervention is not possible. However, the statistician will be blinded for analysis, and microbiology laboratory staff will be blinded when culturing swab growth. The infectious diseases consultant will also be blinded to treatment allocation when apportioning infection outcomes.

#### Procedure for unblinding

Not applicable. There will be no instances where it would be necessary to unblind the statistician, laboratory staff or infectious diseases consultant.

### Data collection and management

#### Plans for assessment and collection of outcomes

**Fig. 1:**
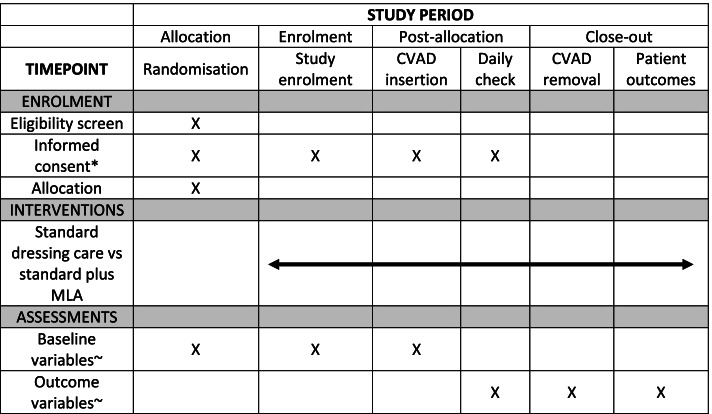
SPIRIT figure

Patient demographic and CVAD insertion data will be collected by research staff at the time of patient enrolment (see Table 1). Research staff will perform daily assessments to collect data on the primary and secondary outcomes, protocol adherence and adverse events (see Fig. 1). To ensure accurate assessment of outcomes related to site complications and adverse skin events, a convenience sample of patients (*n* = 8 per group) will undergo inter-rater reliability assessments of site and skin complications. Research staff will also collect timing data for dressing changes (*n* = 10 per group) to inform cost estimates (see Secondary Outcome 13).

**Table 1:** Data collection

Study enrolment	CVAD insertion	Daily check	CVAD removal	48 h post-CVAD removal
Age Weight Height APACHE II Score Skin type • Very fair, fair, medium, olive, etc. Skin integrity • Good, fair, poor Date of hospital and ICU admission ICU admission type • Planned, emergent, IHT Primary reason for ICU admission • Medical, surgical, other Number of comorbidities Current infections • Blood, urinary, respiratory, etc. Current wounds • Yes, no	CVAD insertion site • Left or right IJ Placement of CVAD in IJ vein • High, mid, low Angle of CVAD lumens • Down, up, horizontal Number of CVAD lumens Place of CVAD insertion • ICU, OT, etc. Antimicrobial-impregnated CVAD • Yes, no Inserter type • ICU registrar, ICU consultant, anaesthetic registrar, etc. Number of insertion attempts Technologies used during insertion • Ultrasound, x-ray, ECG, etc. Hair clipped prior to CVAD insertion • Yes, no Dressing site • Neck, chest, other Diaphoretic at insertion site • Yes, no Facial hair at insertion site • Yes, no Dressing and securements in addition to ‘standard care’ • Sutures, bordered transparent dressing, tissue adhesive, etc. Date and time of first MLA application Staff/patient satisfaction with dressing application • 0 = not at all satisfied, 10 = completely satisfied	Date/time of check Reason why check not able to be completed • Patient not available, etc. Is randomised dressing still in situ • Yes, no Dressing site • Neck, chest, other Dressing and securements in situ • Sutures, bordered transparent dressing, tissue adhesive, etc. Condition of current dressing • Clean, dry, intact, lifting at edges, etc. How many edges of the dressing are lifting or requiring reinforcement Complications at CVAD insertion site • Redness, purulent discharge, pain, etc. Has the patient mobilised, been diaphoretic, restless/agitated • Yes, no Signs and symptoms of skin injury/reaction • Pain, itch, redness, rash, papules, stripping, blister, etc. Suspected cause of MARSI or skin injury/reaction Extent of redness, rash or maceration if applicable Positive bloodstream infections since last daily check • Yes, no Number of administration sets What is the CVAD currently being used for • Fluids, blood products, CVP monitoring, blood sampling, etc. Dressing changes since last daily check • Date/time, reason for dressing change, staff/patient satisfaction, etc.	Date/time of CVC removal Reason for CVC removal • Treatment complete with/without complications, patient deceased, routine replacement, etc. Complications at time of CVC removal • Occlusion, unable to aspirate, accidental removal, suspected infection, MARSI, etc. Signs and symptoms of skin injury/reaction • Pain, itch, redness, etc. Extent of redness, rash or maceration if applicable Pain, tenderness, redness, swelling at CVC insertion site Patient mobility at time of CVC removal • Independent, requires assistance, bed-bound, etc. IV antibiotics administered during CVC dwell • Yes, no Patient diagnosed with delirium • Yes, no Staff/patient satisfaction with dressing removal • 0 = not at all satisfied, 10 = completely satisfied	Was the patient alive at 48 h after CVC removal? • Yes, no Date/time of death SAEs Results of CVC tip culture Results of CVC insertion site swabs Results of blood cultures

*APACHE* Acute Physiology and Chronic Health Evaluation, *CVAD* central venous access device, *CVP* central venous pressure, *ECG* electrocardiogram, *ICU* intensive care unit, *IHT* inter-hospital transfer, *IJ* internal jugular, *MARSI* medical adhesive related skin injury, *MLA* Mastisol liquid adhesive, *OT* operating theatre, *SAE* serious adverse event

At the time of CVAD removal, research staff will collect procedural data, in addition to complications and treatment summary data. If able, patient reported satisfaction (see Secondary Outcome 14) will also be collected, in addition to a convenience sample of skin swabs (*n* = 10 per group) to assess skin colonisation under dressings (see Secondary Outcome 15). Patient outcome and adverse event data will be collected at 48 h after CVAD removal.

#### Plans to promote participant retention and complete follow-up

This study will have dedicated research staff every business day to collect relevant follow-up data for each patient. Clinical staff providing care to enrolled patients over the weekend will be provided education about the study protocol and adequate resources (i.e. MLA if applicable) for dressing changes over the weekend. Research staff will retrospectively collect as much data as possible from the patient’s medical notes and a study-specific bedside data collection log (documenting number of and reason for dressing changes) to complete the weekend data collection as fully as possible.

### Data management

Data will be entered either on to a hard copy data collection form and then transposed into an online Research Electronic Data Capture (REDCap) database [23, 24] or directly entered into the REDCap database. Each site will keep re-identifiable logs of all screened and recruited patients, in addition to an ‘investigator file’ (either electronic or hard copy) in line with ICH Good Clinical Practice requirements.

### Confidentiality

Patient confidentiality will be maintained at all times. Only research staff at each site will have access to identifiable patient information. All data entered into the REDCap database will be de-identified and only re-identifiable at the recruiting site using local screening and recruitment logs. Upon trial completion, only the statistician, project manager and local site principal investigators will have access to the de-identified data once exported from REDCap.

### Plans for collection, laboratory evaluation and storage of biological specimens in this trial and future use

Skin swabs will be collected from the area immediately surrounding the CVAD insertion site to assess micro-organism colonisation in a convenience sample of *n* = 10 per group occurring at either CVAD removal or dressing change if the CVAD has dwelled for three or more days. To do this, a sterile dry swab moistened with 0.9% saline will be firmly moved in a twisting back and forwards motion across the area immediately surrounding the CVAD insertion site. The swab will then be placed in a sterile container and transported to a nearby microbiology laboratory to be qualitatively and quantitatively cultured as per standard practice. After analysis, the swabs will be destroyed.

### Statistical methods

#### Statistical methods for outcomes

All randomised patients will be analysed by intention to treat, except for those patients whose CVAD insertion is cancelled/failed or who withdraw consent. Continuous data will be reported as means (standard deviation) or median (interquartile limits), as appropriate. Categorical data will be presented as frequency (percentage). The primary outcome, dressing failure, will be investigated using logistic regression with ‘treatment’ as the main effect. Incidence rates of dressing failure, skin colonisation and CLABSI with 95% confidence intervals will summarise the effectiveness of each intervention, and Poisson regression will be used to test for group differences. Kaplan-Meier survival curves (with log rank Mantel-Cox test) will compare dressing failure over time. Other secondary clinical outcomes will be compared between groups with appropriate parametric or non-parametric techniques. *P* values < 0.05 will be considered significant.

#### Interim analyses

None planned.

#### Methods for additional analyses

##### Inter-rater reliability

Inter-rater reliability of the daily check site assessment (site complications and evidence of MARSI) will be completed by two research staff at one time point for each of the selected patients using a specific data collection form. Inter-rater reliability will be measured using proportions of specific agreement and by Cohen’s kappa.

##### Cost analysis

Costs between groups will be analysed according to the nursing time taken to conduct dressing changes costed against standard hourly registered nurse wage rates at that site, in addition to cost of resources used as per hospital stores. Costs of treating complications will be based on standard local Diagnosis Related Groups and published estimates.

#### Methods in analysis to handle protocol non-adherence and missing data

Prior to analysis, data will be cleaned and attempts at locating missing data will be made. Missing data that is unable to be found will not be imputed prior to analysis. In addition to intention-to-treat analyses, per protocol analyses will also be completed to address protocol non-adherence.

#### Plans to give access to the full protocol, participant level data and statistical code

The study has been prospectively registered with the Australian New Zealand Clinical Trials Registry (ACTRN12621001012864), and the protocol will be published in an open access, peer-reviewed journal before the end of patient recruitment.

The datasets generated during and/or analysed during the current study are available from the corresponding author on reasonable request.

### Oversight and monitoring

#### Composition of the coordinating centre and trial steering committee

The coordinating centre is responsible for concept inception, study design, funding acquisition and ethical conduct of the trial. The coordinating centre comprises of the chief investigator and associate investigators, including the project manager. There is no formal steering committee for this study.

#### Composition, role and reporting structure of the data monitoring committee

Not applicable.

#### Adverse event reporting and harms

Enrolled patients will be monitored and treated for untoward medical occurrences in line with standard clinical care. Therefore, only adverse events which the treating clinicians believe are associated with the intervention will be reported.

In this trial, the following will be considered as serious adverse events (SAEs):

- Death
- CLABSI
- MARSI

All SAEs from randomisation to 48 h after removal of the CVAD will be recorded on REDCap and reported to the coordinating centre within 24 h.

The minimum information to report will include:

- Patient study number
- Nature of the event
- Commencement and cessation of event
- The principal investigator’s assessment of the relationship between the study intervention and the event (not related, possibly related, probably related)
- Whether treatment was required for the event and what treatment was administered

It is the responsibility of each site’s principal investigator to inform the chief investigator and project manager of all SAEs which occur at their site. Copies of reports and correspondence to and from the reviewing HREC and research governance will also be sent to the coordinating centre. The project manager will be responsible for reporting all SAEs to the reviewing HREC and alerting other participating sites of the SAE if required.

#### Frequency and plans for auditing trial conduct

The project manager will undertake quality checks for allocation integrity and monitor 100% source data verification for the first patient per site, consent forms, primary outcome and a random 5% of other data for all patients. The project manager will also conduct regular remote monitoring on the REDCap database and regular data cleaning to ensure the integrity of the study data. Data queries will be compiled and sent to each participating site at regular intervals throughout the study and as part of final data cleaning.

#### Plans for communicating important protocol amendments to relevant parties (trial participants, sites, HRECs)

The project manager will be responsible for communicating protocol amendments to the reviewing HREC and recruiting sites. The project manager will also be responsible for ensuring amendments and reports are forwarded by research staff to Research Governance at each site. The project manager will notify research staff at each recruiting site if amendments or new data have the potential to impact patients, who will then inform all relevant participants.

### Dissemination plans

Locally, results will be presented at hospital seminars including the clinical departments which participate in the trial, and at annual hospital symposiums. Results will be published in a relevant peer-reviewed journal with a wide readership. Results will also be disseminated through conference presentations at local and international nursing and medical assemblies. The investigators are members of professional organisations and bodies including infusion nursing and infection prevention and will use their professional networks to further highlight trial results.

Authorship will be determined as per the National Health and Medical Research Council Authorship Guidelines [25].

## Discussion

The aim of this trial is to assess the effectiveness of MLA, compared to ‘standard’ dressing care, in improving dressing adhesion and reducing dressing changes in internal jugular CVADs. This trial has several strengths and limitations. A strength of the study is its randomised design which minimises bias and confounding factors thereby increasing the reliability of the results. However, a limitation of this study is the inability to double-blind randomisation allocation due to the nature of the intervention, which may introduce performance bias. Another strength of this protocol is the requirement for daily checks of the central line dressing to ensure accurate data collection and monitoring for serious adverse events. This is particularly relevant as there is very limited pre-existing evidence of skin reactions to and effectiveness of MLA. However, daily checks will not be able to be carried out in person on weekends due to staffing limitations. Nonetheless, this study will be the first randomised controlled trial to assess the clinical and cost effectiveness of MLA and, as such, will contribute much needed evidence on strategies to reduce CVAD dressing failure in critically ill patients.

## Trial status

Current protocol: Version 1.0, dated 13 May 2021

Date recruitment began: 02 September 2021

Anticipated date of recruitment completion: 01 September 2022

## Data Availability

To maintain data privacy, investigators at each site will only have access to the data collected at their respective site. The project manager, statistician and chief investigator will be the only people with access to the final de-identified trial dataset. External requests for access to the final dataset may be made to the corresponding author after results publication.

## Abbreviations

CDC: Centres for Disease Control and Prevention
CLABSI: Central line associated blood stream infection
CVAD: Central venous access device
HREC: Human research ethics committee
ICH: International Council for Harmonisation of Technical Requirements for Pharmaceuticals for Human Use
MARSI: Medical adhesive-related skin injury
MLA: Mastisol liquid adhesive
NHSN: National Healthcare Safety Network
RCT: Randomised controlled trial
REDCap: Research Electronic Data Capture
SAE: Serious adverse event

## References

[1] Ullman AJ, Marsh N, Mihala G, Cooke M, Rickard CM (2015). Complications of central venous access devices: a systematic review. Pediatrics..

[2] Rickard C, Edwards M, Spooner A, Mihala G, Marsh N, Best J (2016). A 4-arm randomized controlled pilot trial of innovative solutions for jugular central venous access device securement in 221 cardiac surgical patients. J Crit Care..

[3] Tuffaha HW, Marsh N, Byrnes J, Gavin N, Webster J, Cooke M (2019). Cost of vascular access devices in public hospitals in Queensland. Aust Health Rev..

[4] Corley A, Marsh N, Ullman AJ, Rickard CM (2017). Tissue adhesive for vascular access devices: who, what, where and when?. Br J Nurs..

[5] Napalkov P, Felici DM, Chu LK, Jacobs JR, Begelman SM (2013). Incidence of catheter-related complications in patients with central venous or hemodialysis catheters: a health care claims database analysis. BMC Cardiovasc Disord..

[6] Rosenthal VD, Guzman S, Migone O, Crnich CJ (2003). The attributable cost, length of hospital stay, and mortality of central line-associated bloodstream infection in intensive care departments in Argentina: a prospective, matched analysis. Am J Infect Control..

[7] Periard D, Monney P, Waeber G, Zurkinden C, Mazzolai L, Hayoz D (2008). Randomized controlled trial of peripherally inserted central catheters vs. peripheral catheters for middle duration in-hospital intravenous therapy. J Thromb Haemost..

[8] O'Grady NP, Alexander M, Burns LA, Dellinger EP, Garland J, Heard SO (2011). Guidelines for the prevention of intravascular catheter-related infections. Am J Infect Control..

[9] Infusion Nurses Society (2021). Infusion therapy standard of practice (8^th^ ed.). J Infus Nurs.

[10] Ullman AJ, Cooke ML, Mitchell M, Lin F, New K, Long DA (2015). Dressings and securement devices for central venous catheters (CVC). Cochrane Database of Syst Rev..

[11] Naimer SA, Temira F (2004). Evaluation of techniques for intravenous catheter and tubing fixation. Mil Med..

[12] Ryder M, Duley C (2017). Evaluation of compatibility of a gum mastic liquid adhesive and liquid adhesive remover with an alcoholic chlorhexidine gluconate skin preparation. J Infus Nurs..

[13] Broadhurst D, Moureau N, Ullman AJ (2017). Management of central venous access device-associated skin impairment: an evidence-based algorithm. J Wound Ostomy Continence Nurs..

[14] Ullman AJ, Mihala G, O’Leary K, Marsh N, Woods C, Bugden S (2019). Skin complications associated with vascular access devices: a secondary analysis of 13 studies involving 10,859 devices. Int J Nurs Stud..

[15] Timsit J-F, Bouadma L, Ruckly S, Schwebel C, Garrouste-Orgeas M, Bronchard R (2012). Dressing disruption is a major risk factor for catheter-related infections. Crit Care Med..

[16] McCord J, Niehaus S (2016). Improving adhesion of internal jugular dressings in the intensive care unit. JAVA..

[17] The Prince Charles Hospital. Central venous line/PICC line/vascath equipment required for insertion. 2021.

[18] South Western Sydney Local Health District. Policy directive: Central venous access device (CVAD) post insertion management (SWSLHD_PD2018_011). 2018.

[19] Health Q (2019). Recommendations for the prevention of infection in intra-vascular devices.

[20] National Healthcare Safety Network. Patient safety component manual. Chapter 4: Bloodstream infection event (Central line-associated bloodstream infection and non-central line associated bloodstream infection). 2021. Access from https://www.cdc.gov/nhsn/pdfs/pscmanual/pcsmanual_current.pdf

[21] National Healthcare Safety Network (2021). CDC/NHSN surveillance definitions for specific types of infections.

[22] Fumarola S, Allaway R, Callaghan R, Collier M, Downie F, Geraghty J (2020). Overlooked and underestimated: medical adhesive-related skin injuries. J Wound Care..

[23] Harris PA, Taylor R, Minor BL, Elliott V, Fernandez M, O'Neal L (2019). The REDCap consortium: building an international community of software platform partners. J Biomed Inform..

[24] Harris PA, Taylor R, Thielke R, Payne J, Gonzalez N, Conde JG (2009). Research electronic data capture (REDCap)--a metadata-driven methodology and workflow process for providing translational research informatics support. J Biomed Inform..

[25] National Health and Medical Research Council. Authorship: a guide supporting the Australian Code for the Responsible Conduct of Research. National Health and Medical Research Council, Australian Research Council and Unversities Australia; 2019. Accessed from https://www.nhmrc.gov.au/sites/default/files/documents/attachments/Authorship-Guide.pdf

